# Sub-Optimal Treatment of Bacterial Biofilms

**DOI:** 10.3390/antibiotics5020023

**Published:** 2016-06-22

**Authors:** Tianyan Song, Marylise Duperthuy, Sun Nyunt Wai

**Affiliations:** 1 Department of Molecular Biology, Umeå University, Umeå S-90187, Sweden; tianyan.song@umu.se (T.S.); marylise.duperthuy@umu.se (M.D.); 2 The Laboratory for Molecular Infection Medicine Sweden (MIMS), Umeå University, Umeå S-90187, Sweden; 3 Department of Clinical Microbiology, Umeå University, Umeå S-90185, Sweden

**Keywords:** biofilm development, sub-optimal treatment, antibiotic tolerance, anti-biofilm agents

## Abstract

Bacterial biofilm is an emerging clinical problem recognized in the treatment of infectious diseases within the last two decades. The appearance of microbial biofilm in clinical settings is steadily increasing due to several reasons including the increased use of quality of life-improving artificial devices. In contrast to infections caused by planktonic bacteria that respond relatively well to standard antibiotic therapy, biofilm-forming bacteria tend to cause chronic infections whereby infections persist despite seemingly adequate antibiotic therapy. This review briefly describes the responses of biofilm matrix components and biofilm-associated bacteria towards sub-lethal concentrations of antimicrobial agents, which may include the generation of genetic and phenotypic variabilities. Clinical implications of bacterial biofilms in relation to antibiotic treatments are also discussed.

## 1. Introduction

Microorganisms, such as bacteria and fungi, can grow in free-living (planktonic) or in cell aggregates (biofilm). These biofilms are highly organized communities of microbes consisting of one or more species surrounded by an extracellular matrix (ECM). The composition of the ECM varies depending on the organisms within the biofilm, but in general it includes microbial-derived complex polysaccharides, proteins, lipids and nucleic acids [1]. In addition to microbial components, host factors such as fibrin, platelets and immunoglobulins may also be integrated into the ECM [2]. The phenomenon of biofilms has been observed for several hundred years, with the earliest description by Anthony van Leeuwenhoek (1632–1723) [3]. In the 1970s, Jendresen and Hoiby were among the first to recognize the importance of biofilms in medicine through their observations of acquired dental pellicles and aggregates of *Pseudomonas aeruginosa* (*P. aeruginosa*) bacteria in sputum and lung tissues from cystic fibrosis patients [3]. The term biofilm, originally used in technical and environmental microbiology, was introduced into medicine in 1985 by Costerton, following observations that *Staphylococcus aureus* (*S. aureus*) formed biofilms on cardiac pacemaker leads [3,4,5]. These revolutionary observations have stimulated research on how biofilms are found during infections and their molecular features. Over the past 40 years, the field has accumulated abundant evidence to support the concept that bacterial biofilms are important during the pathogenesis of infection. The U.S. National Institutes of Health estimates that more than 80% of human microbial infections have biofilm origin [6]. A particular concern is that biofilm-forming bacteria are often exposed to sub-lethal doses of antibiotics because the biofilm structure generates a concentration gradient from the surface to the interior of the biofilm. It was also suggested that, in the soft tissues, e.g., the intestine and the lungs, the pathogens or microbiota might only be exposed to sub-minimum inhibitory concentration (MIC) levels of antibiotics [7,8]. The sub-lethal concentration of antibiotics might influence on the evolution of antibiotic resistance and tolerance. In this review, we summarize current knowledge about bacterial responses to sub-lethal concentrations of antibiotics and the related implications of biofilm formation and clinical consequences.

## 2. Biofilm Formation and Related Infections

Biofilm formation enables bacteria, otherwise seen as single-cell living organisms, to participate in multicellular lifestyle, in which “group behavior” helps survival of bacteria in adverse environmental conditions. Bacterial biofilm formation and maturation consist of reversible and irreversible stages and include several conserved and/or species-specific bacterial factors [9]. The first step involves bacterial adherence or attachment to a substratum, which is influenced by several factors including motility, chemotaxis, extracellular adhesive appendages and secreted adhesins. The second step includes biofilm maturation. Surface contact during the first step triggers stimuli leading to changes in expression of bacterial genes implicated in extracellular matrix formation. Within the mature biofilm, the bacterial community actively shares products that play an important role in maintaining biofilm architecture and providing a favorable environment for survival of resident bacteria. The third step involves escape from the matrix, so-called dispersing. Biofilm dispersal can be influenced by several factors including alterations in nutrient availability, oxygen fluctuation, toxic products, and other stress-inducing conditions [10,11,12,13].

Depending on whether there is an implanted device involved in the biofilm formation, biofilm infections are divided into two major groups: device-related and tissue-related. Numerous medical devices have been found to be associated with the occurrence of biofilm infections, including orthopedic alloplastic devices [14,15], indwelling urinary catheters or urethral stents [16,17], intravenous catheters [18], vascular prostheses [19], cardiac pacemakers and prosthetic heart valves [4,16,20], endotracheal tubes [21], cerebrospinal fluid shunts [22], peritoneal dialysis catheters [23], biliary tract stents [24], intrauterine devices [25,26], contact lenses [27], tissue fillers [28,29], and dentures [30]. Biofilm infections that are not associated with implanted devices include the chronic airway infection commonly seen in patients with cystic fibrosis [31], chronic obstructive pulmonary diseases [32], tuberculosis [33], chronic wound infections [34], chronic otitis media [35], and chronic sinusitis [36]. In addition to antibiotic therapy, removal of the underlining cause (e.g., infected foreign body and necrotic tissue debris) is most often considered necessary for successful treatment of biofilm infection [37].

## 3. Antimicrobial Penetration of Biofilms 

The inability of antibiotics to penetrate through the biofilm is one of the important factors contributing to the capacity of bacterial biofilms in antibiotic tolerance. Bacteria at the surface of the biofilm can encounter a lethal concentration of a particular antimicrobial agent, whereas bacteria at the center of the biofilm, which are protected by the matrix, might be exposed to a low or sub-inhibitory concentration. The penetration rates of antibiotics through biofilms are dependent not only on the bacterial specie(s) forming the biofilm but also on the antibiotic used. For instance, ciprofloxacin can quickly and completely penetrate through *Klebsiella pneumoniae* (*K. pneumoniae*) biofilms, whereas ampicillin cannot [38]. It was also demonstrated that ampicillin could efficiently penetrate the biofilms formed by β-lactamase mutant of *K. pneumoniae* although the bacterium remained resistant to ampicillin. Similarly, ciprofloxacin and tetracycline are capable of penetrating non-mucoid *P. aeruginosa* and *Escherichia coli* (*E. coli*) biofilms, respectively [39,40,41]. Recently, it was demonstrated that the positively charged antibiotic tobramycin was sequestered near the biofilm surface and did not efficiently penetrate the biofilm, whereas ciprofloxacin penetration was efficient. The authors demonstrated that the positively charged antibiotic ionically interacted with negatively charged biofilm matrix components causing reduced penetration [40]. Further, this interaction could be inhibited by addition of excess cations before addition of tobramycin, substantially increased the penetration of tobramycin into the biofilm. Diffusion and penetration experiments have also been performed for biofilms formed by Gram-positive bacteria. At clinically relevant concentrations, antibiotic penetration within the biofilms of *S. aureus* is strain-dependent. Among biofilms formed by seven different isolates, penetration rates ranged from 0.6% to 52% for delafloxacin, 0.2% to 10% for daptomycin, and 0.2% to 1% for vancomycin. Penetration of delafloxacin through biofilm is inversely related to the proportion of polysaccharide in the matrix. Addition of norspermidine and norspermine, which led to disassembling of the biofilm matrix, increased delafloxacin penetration and efficacy, while only modestly improving daptomycin penetration and efficacy [42,43]. A recent study by Doroshenko *et al*. demonstrated that pre-exposure of a *Staphylococcus epidermitis* (*S. epidermitis*) strain with a sub-lethal concentration of vancomycin increased the tolerance of the biofilm to vancomycin by reducing its penetration within the biofilm [44].

For the enhancement of antimicrobial penetration into the biofilms, several approaches have been described by different research groups. Biosurfactants are amphiphilic compounds produced by microbes or plants and may exhibit strong antimicrobial, anti-adhesive, and anti-biofilm properties making them good candidates for applications used to combat biofilm-related infections. A recent study showed that biosurfactants extracted from *Lactobacilli* have antibacterial properties against multidrug-resistant *Acinetobacter baumannii*, *E. coli* and *S. aureus* strains [45]. The lipopeptide biosurfactant isolated from *Bacillus licheniformis,* when tested in combination with antibiotics, led to enhancement of antibiotic penetration in biofilms formed by *E. coli* CFT073 [46]. Pamp *et al*. [47] provided evidence that the metabolically active bacterial cells in *P. aeruginosa* biofilms were able to develop tolerance to the antimicrobial peptide colistin whereas the biofilm cells with low metabolic activity were killed by colistin. In contrast, conventional antibiotics such as ciprofloxacin and tetracycline killed the metabolically active biofilm cells, although the bacterial population in the biofilm with low metabolic activity survived the treatment. Moreover, the authors demonstrated that targeting the two physiologically distinct bacterial subpopulations in the biofilm by combined antimicrobial treatment with either ciprofloxacin and colistin or tetracycline and colistin almost completely eradicated all biofilm cells. Recently, Cheow *et al*. demonstrated that antibiotic-loaded polymeric nanoparticles can efficiently kill biofilm cells [48]. In addition, inactivation of efflux systems by efflux pump inhibitors was reported to abolish bacterial biofilm formation and to enhance antimicrobial activity against biofilms [49].

## 4. Biofilm Response to Sub-Lethal Concentrations of Antibiotics

The first study demonstrating the link between sub-MIC antibiotic and biofilm formation *in vitro* was reported in 1988 [50]. In this study, rifampicin at a concentration corresponding to 1/4 of the MIC could induce biofilm formation in one of the three *S. epidermidis* strains. Eight other antibiotics at 1/4 MIC had either no effect or reduction of biofilm formation. Since then, several studies have been carried out to study the effect on biofilm formation by low doses of antibiotics. A majority of the studies found that low doses of antibiotics induce biofilm formation, as Kaplan described in a review article [51].

The capacity of sub-lethal levels of antibiotics to modulate bacterial virulence *in vitro* has recently been brought to light, raising concerns over the appropriateness of low-dose antibiotic therapies. In fact, besides virulence regulation, bacterial stress response, motility and biofilm formation are all affected by exposure of bacteria to sub-lethal concentrations of antibiotics targeting diverse cellular processes [51,52,53,54]. The bacterial cells within a biofilm are significantly less sensitive to many antimicrobials targeting metabolic pathways since these cells are generally less metabolically active than planktonic cells [55]. Bacterial cells within a biofilm may also develop into persister cells, which are transiently antibiotic tolerant without the concomitant genetic changes seen in antimicrobial resistance [56]. Both the cell density and the age of a biofilm influence its antimicrobial susceptibility [57]. In addition, composition of the growth medium may also play a role. For example, young biofilms formed by *E. coli* in Luria-Bertani medium can be totally dispersed by kanamycin, whereas the addition of glucose drastically increases kanamycin resistance [58]. However, no role has been observed for the size or chemistry of the antimicrobial agent, the substratum, nor the microbial species composition on the level of antibiotic tolerance in biofilms [57]. Currently, extensive research is being conducted to identify anti-biofilm compounds to compensate the negative effect of sub-inhibitory concentration of antibiotics. For instance, limonene, a cyclic terpene found in the rind of citrus fruits, displays anti-biofilm potential against species of the genus *Streptococcus*. Based on surface coating-assays and microscopy of the biofilm structures, the authors suggest that limonene possibly acts by inhibiting bacterial adhesion to surfaces [59].

### 4.1. Phenotypic Changes

Several phenotypic changes have been observed when biofilms are exposed to sub-lethal concentrations of antibiotics, including biofilm structure, cell morphology, bacterial growth rate, production of extracellular DNA and release of bacterial membrane vesicles.

#### 4.1.1. Alteration of Biofilm Structure and Cell Morphology

Treatment with sub-lethal concentrations of antibiotics can lead to alteration in the structure of the biofilm and/or bacterial cell morphology. Exposure of *K. pneumoniae* to a sub-lethal concentration of carbapenem caused modifications in size and shape with the appearance of rounded cells through RpoS-dependent regulation [60]. Following 24 h of imipenem treatment, there was significant cell shortening, while treatment with meropenem and doripenem led to significant cell lengthening compared to that of cells in an untreated biofilm. Moreover, material resembling cellular debris was accumulated after antibiotic exposure. The morphological alteration of the cells was reversible and induced no significant change in viability. The morphology of cells can also be altered by exposing planktonic cells to a sub-lethal concentration of antibiotic, causing a direct effect on the ability to form biofilm. For instance, treating *E. coli* cells with a sub-inhibitory concentration of piperacillin or a combination of piperacillin and tazobactam resulted in filamentation [61,62]. Generation of the filamentous form of *E. coli* cells was accompanied by the inhibition of virulence factors, biofilm formation, cell surface hydrophobicity and motility, concordant with a reduction in the pathogenicity observed for the filamentous *E. coli* in a murine model of intra-abdominal infection [62]. Besides antibiotics, other compounds at sub-lethal concentrations can also alter the morphology of cells in the biofilm. For instance, a biofilm of *Streptococcus mutans* treated with a sub-lethal concentration of *Curcuma xanthorrhiza* extract, which contains xanthorrhizol, showed morphological changes in the cell wall and membrane, an uneven surface and contour deformation [63].

#### 4.1.2. Reduced Growth of Bacteria

Metabolic activity of bacteria in biofilms depends on their localization within the biofilm and is influenced by their access to nutrients. Bacteria at the surface of a biofilm are the most metabolically active cells due to greater access to nutrients and they are more sensitive to all types of antimicrobials. Cells in the middle of the biofilm are essentially non-growing but still alive and may acquire tolerance to some agents. Such cells may exhibit active membrane potential and capacity for ATP generation along with a lower capacity for transcription and translation. These cells do not exhibit DNA replication, cell wall synthesis, or the normal balance of proteins required for division, thus become insensitive to β-lactam antibiotics, which target on cell wall synthesis. Deepest in the biofilm, bacteria are in an inactive and non-growing state lacking any catabolism or anabolism. They cannot maintain a membrane potential and thus might become insensitive to aminoglycoside antibiotics, which require active transport to reach their intracellular targets. Therefore, bacteria deepest in the biofilm can potentially remain dormant and become tolerant to a wide range of antimicrobial agents. Dormant cells have acquired protective modifications, including alteration of membrane lipid and porin composition to reduce permeability, hibernation of ribosomes, inhibition of transcription and replication machineries, and deployment of enzymes that protect against oxidative stress without consuming ATP (e.g., catalase) [57]. Consequently, these persister cells can provide a source for bacterial re-growth following antibiotic withdrawal.

Because of nutrient starvation in mature biofilms, slow bacterial growth has been observed in these communities. It has been known for decades from studies of planktonic bacteria in batch cultures that a transition from exponential to slow or no growth is usually associated with an increase in tolerance to antibiotics [64,65,66]. This mechanism is therefore highly dependent on the capacity of the nutrients to diffuse into the biofilm. Such diffusion also depends on the nature of the nutrient and on the species involved in the biofilm [67,68]. The lowered metabolic activity of at least some biofilms might be partly responsible for their enhanced resistance to treatment with antibiotics, especially for antibiotics that target on growth factors in planktonic bacteria. Because the above-mentioned studies were performed with planktonic bacteria, it remains unclear how dormancy and persister cells are modulated in biofilms upon exposure to sub-lethal dose of antibiotics.

#### 4.1.3. Induction of Extracellular DNA

The role of extracellular DNA (eDNA) in biofilm establishment, maintenance and perpetuation has been reviewed recently [69,70,71]. eDNA is involved in all stages of biofilm formation, from initial bacterial adhesion to maintenance of the biofilm’s structural integrity. eDNA adsorbs to the surface of bacterial cells and forms loop extension, which facilitates its adhesion to the substratum. Due to its acid-base interactions and amphiphilic properties, eDNA also participates in the second step of bacterial adhesion to the substratum, *i.e.*, the biologically active adhesion involving adhesins. Furthermore, eDNA is important for maintenance of the integrity of the biofilm. Consequently, DNase treatment can disintegrate biofilms, and addition of eDNA to bacterial cultures increases the aggregation of bacteria such as *E. coli* [72]. However, the importance of eDNA in biofilm stability seems to be more influential in the early stages of biofilm development, suggesting that other matrix components replace or complement the role of the eDNA in the mature biofilms.

The eDNA found in biofilms provides an abundant substrate for naturally occurring transformation, one of the alternative mechanisms resulting in horizontal gene transfer. It is well established that gene transfer is enhanced in biofilms in comparison to that occurring in environmental planktonic conditions. Moreover, bacterial competence is increased in the presence of eDNA, and the release of DNA is an active process in the induction of competence [70]. It is also interesting to note that in multispecies biofilms it is common that one species is lysed because of interspecies competition, leading to the release of its DNA. The eDNA liberated is therefore available for horizontal gene transfer to competent cells in the biofilm. This sharing of genetic material may lead to environmental adaptations including antibiotic resistance [73]. eDNA also has a role in antimicrobial resistance via direct binding to the cationic antimicrobials, including the human antimicrobial peptide β-defensin-3 [74] and to aminoglycoside antibiotics [75]. Furthermore, eDNA can act indirectly in antimicrobial resistance by triggering the expression of genes involved in resistance. Indeed, DNA can bind to divalent cations, which are used as signal molecules for two-component systems such as PmrAB or PhoPQ, necessary to regulate expression of virulence and antimicrobial resistance genes [76]. Interestingly, *S. epidermitis* biofilms exposed to sub-lethal concentrations of vancomycin exhibit a higher amount of eDNA [44]. The authors demonstrated that eDNA and vancomycin underwent spontaneous chemical interaction that blocked antimicrobial activity. This binding also explains the limited penetration of vancomycin through the vancomycin-exposed biofilm, and thus, increased resistance towards vancomycin.

#### 4.1.4. Induction of Bacterial Membrane Vesicles (BMVs) Formation

Using planktonic bacteria, several studies have shown that release of BMVs can be induced in response to sub-lethal concentrations of antimicrobial agents. In *Shigella dysenteriae*, mitomycin C was found to promote formation of BMVs through increased vesiculation of the bacterium and concomitant promotion of BMV-mediated release of Shiga toxin [77]. In *Vibrio cholerae* (*V. cholerae*), antimicrobial peptides have been shown to activate the envelope stress-response alternative sigma factor σ^E^ through an OmpU-mediated signaling pathway [78,79]. Following activation of σ^E^, production of a small RNA (VrrA) is induced in *V. cholerae*, which leads to increased production of BMVs through repression of OmpA, an outer membrane protein important in maintaining cell membrane integrity [80]. The BMVs were found to protect the bacterial cells from antibiotic stress when challenged with lethal concentrations of membrane perturbing peptides including polymyxin B, colistin, and melittin [81,82]. Moreover, BMVs play a role in biofilm formation and maintenance such as mediating adherence, delivering material and competing for growth factors. BMVs were found to be important components of *Helicobacter pylori* biofilms, and the addition of BMVs to a *Helicobacter* planktonic culture triggered the biofilm formation. A recent proteomic analysis of BMVs obtained from planktonic growth and in biofilm of *P. aeruginosa* revealed that drug-binding proteins (notably efflux proteins) were more concentrated in the biofilm BMVs suggesting a possible drug-sequestering effect by vesicle proteins [83,84]. Interestingly, a recent study showed that *S. aureus* membrane vesicles carried biologically active BlaZ, a β-lactamase protein, and these vesicles make it possible for other ampicillin-susceptible Gram-negative and Gram-positive bacteria to survive in the presence of ampicillin. It provides evidence of the important role of *S. aureus* vesicles in antibiotic resistance, which allows the polymicrobial community such as in a biofilm to continue to evolve and prosper against antibiotics [85]. Despite intense research on modulation of BMVs release from planktonic bacteria in response to antibiotics, there is little or no information about influence of antibiotics on BMVs production from biofilm-associated bacteria.

### 4.2. Alteration of Signaling Networks

Biofilm formation is tightly controlled by regulatory networks to enable bacteria to respond to various environmental cues. Such regulatory networks include transcriptional factors, alternative RNA polymerase sigma factors, starvation stress response, SOS response, oxidative stress response, quorum sensing, and signaling molecules such as c-di-GMP. Several studies have shown that these molecular networks are altered by exposure to sub-lethal concentrations of antibiotics.

#### 4.2.1. Starvation Stress Response

Earlier studies demonstrated that one of the most important causes of starvation-induced antibiotic tolerance *in vivo* is biofilm formation during chronic infections [86,87]. Starvation in biofilms was suggested to be due to nutrient consumption by the bacterial cells present at the periphery of biofilm clusters and by reduced diffusion of nutrient through the biofilm [57]. An elegant study demonstrated that reduced drug target activity or growth arrest *per se* in the biofilm are not responsible for the tolerance of biofilm bacteria, whereas RelA-SpoT mediated starvation-signaling stringent response is required. The authors demonstrated that starvation responses can protect the bacterial community in the biofilm by reducing the production of pro-oxidant metabolites and increasing antioxidant defenses [88].

#### 4.2.2. SOS Response

The SOS response is involved in horizontal gene transfer and adaptation of bacteria potentially leading to the onset of antibiotic resistance in a broad range of bacterial species. SOS response activates the DNA repair systems, such as methyl mismatch repair (MMR) or DNA oxidative repair system. Such mutations usually lead to better adaptation of bacterial clones to the hostile environments. Interestingly, a high number of bacteria inside biofilms exhibit mutation(s) in DNA repair genes (*i.e.*, *mut* genes of MMR), leading to a hypermutator phenotype [89]. The hypermutator can have a selective advantage in the biofilm in terms of competitiveness, ability to form biofilm, antibiotic resistance and persistence. For instance, *P. aeruginosa* mismatch repair system-deficient mutators have enhanced adaptability when grown in biofilms but not as planktonic cells. This advantage is associated with enhanced micro-colony development and increased rates of morpho-phenotypic diversification. Morphotypic variants generated in mutator biofilms also showed increased competitiveness, suggesting a mutator-driven adaptive evolution specific to the biofilm [90]. Moreover, *in vivo* experiments demonstrated that *P. aeruginosa* hypermutators are not only less virulent than the wild-type strain, but also more capable of colonizing mice oropharynx, supporting the hypothesis that the hypermutator has an advantage in persistent biofilm infections [91].

#### 4.2.3. Oxidative Stress Response

The presence of genetic diversity and of physiological heterogeneity in a bacterial population grown in biofilm have been clearly established for several bacterial species [92]. This diversity results in the production of phenotypic variants in colony morphology, motility, pigmentation, biofilm formation capacity, or dissemination ability. An elegant study from Boles and Singh [93] demonstrated that these variants do not result from spontaneous gene mutations or emergence of supermutator, but occur as a response to endogenous oxidative stress. To counterbalance the oxidative stress, bacteria activate the DNA double-strand break repair system and the recA gene, which is essential for recombinational DNA repair. The authors further demonstrated that the DNA break repair system is responsible for a drastically increased proportion of gentamicin-resistant variants in the biofilm in presence of gentamicin at a sub-lethal concentration [93,94].

#### 4.2.4. c-di-GMP Signaling

In recent years, the second messenger cyclic dimeric guanosine monophosphate (c-di-GMP) has evolved as a major regulator of bacterial biofilm formation [95]. The intracellular level of c-di-GMP can be affected by certain antibiotics. Hoffman *et al*. [96] elegantly demonstrated that tobramycin, a commonly used aminoglycoside, at sub-lethal concentration could increase the mass of biofilms formed by *P. aeruginosa* and *E. coli*, and the molecular basis of this effect was through the c-di-GMP signaling. In addition to the activation of biofilm formation, c-di-GMP is also important in the regulation of persister cell formation [97,98]. Reversion of dormancy or persistency by manipulating the c-di-GMP level may serve as a strategy to target the antibiotic tolerance of persister cells.

#### 4.2.5. Quorum Sensing

Quorum sensing (QS) mechanisms are used by bacteria to coordinate gene expression in accordance to the density of their local population. The development of antibiotic-tolerant bacterial colonies within biofilms is found to be coordinated by QS [99]. During chronic *P. aeruginosa* infection of cystic fibrosis (CF), despite antibiotic treatment, high bacterial density was observed in sputum of most patients. The persistence of bacteria was correlated with progressive lung damage [100]. Genetic analysis of *P. aeruginosa* isolates from the airways of several CF patients revealed that a QS transcriptional regulator *lasR* gene was one of the most common targets of mutation in CF isolates [101]. In addition, it was demonstrated that the *lasR* mutation in *P. aeruginosa* resulted in increased beta-lactamase activity that increased resistance to ceftazidime, a widely used beta-lactam antibiotic. Interestingly, the authors found that the *lasR* mutants were more sensitive to fluorophenylalanine. It suggests that LasR-mediated *P. aeruginosa* adaptations to the CF airway could be used as targets for new treatment strategies [102,103]. Recent study indicated a dynamic development of drug-tolerant bacterial subpopulations in *P. aeruginosa* biofilms after treatment with colistin, a last antibiotic available for treatment of drug-resistant Gram-negative bacterial infections. The colistin-tolerant populations were shown to migrate to the top of the dead biofilm by using type IV pili-dependent motility to initiate new biofilm formation via QS-regulated mechanism. The authors highlighted the importance to develop QS- and pilus-inhibitors for functional anti-biofilm chemotherapies [104]. In addition to colistin-tolerance, the appearance of colistin-resistance is rising as demonstrated by the emergence of transmissible, plasmid-mediated colistin resistance encoded by the *mcr-1* gene. The plasmids carrying *mcr-1* have been worryingly observed in an increased number of bacterial strains worldwide including isolates from gut microbiota of diarrhea patients [105,106]. It is thus important to take into consideration both antibiotic tolerant and resistant populations when developing novel therapeutic strategies for eradication of biofilm-associated infections.

## 5. Clinical Implications

The correlation between biofilms and chronic infections is clear [107], as outlined above and exemplified by *P. aeruginosa* infections in cystic fibrosis [108], *E. coli* in urinary tract infections [109] and *S. aureus* in chronic wound infections [110]. The correlation is further supported by the observations that many device-related chronic biofilm infections can be cured upon removal of the infected device [20,111], suggesting that biofilm is the underlying cause. A major challenge in the treatment of biofilm-related infection is the biofilm’s recalcitrance towards antibiotics, which is a problematic mixture of drug-resistance bacterial cells and drug-tolerant persister cells [112]. It is now clear that biofilms can provide an important reservoir for persister cells [87], a subset of biofilm bacteria that do not grow in the presence of antibiotics and arise through efficient gene regulation without undergoing genetic change [113]. When the concentration of antibiotic decreases, persisters will be shed from the biofilm and become planktonic cells that can cause relapse biofilm infection [114,115].

Knowing that persister cells are important in causing recurrence of biofilm infection, the question now is how these persisters can be successfully eradicated. In contrast to resistant cells that carry resistance genes and thus become genetically resistant towards antibiotics, persister cells do not carry resistance genes and therefore are susceptible to antibiotics. This likely explains why many recurrent infections can be resolved by the same antibiotic during the beginning episodes before resistant bacteria arise. Because the increase in age of the biofilm is correlated with higher tolerance towards antibiotics [57], it is important to eradicate persister cells as early as possible during the course of infection. This requires early diagnosis of a biofilm infection and early initiation of adequate antibiotic therapy. Diagnosis of biofilm infection is based on clinical symptoms in combination with laboratory investigations. To determine the effective dose and duration of antibiotic therapy is a challenging task for specialists in infectious diseases and clinical microbiology. MIC is the most commonly used test to estimate the sensitivity of a given bacterium towards a given antibiotic. Because the MIC test is performed *in vitro* using standardized laboratory conditions with planktonic cells, it hardly reflects the *in vivo* situation where it is likely that multi-species communities of bacteria are present together with immunological factors from the host. In fact, studies show that persisters in biofilms are capable of surviving in the presence of very high concentrations of antibiotics, in some cases up to 1000-fold of MIC [114,116]. For the treatment of biofilm infections, concentrations much higher than the MIC are necessary and the recommended doses by guidelines are in general empirical [37], thus it remains unclear whether these concentrations reach a lethal dose for bacteria at the infection site. The pharmacokinetic and pharmacodynamic information on the activities of antimicrobial agents against biofilm-associated bacteria will contribute to better clinical treatment of biofilm-associated infections [8].

What happens after sub-optimal treatment of biofilm infection? Do the bacteria become more pathogenic because of sub-optimal treatment? The answer may be dependent on the type of bacteria, the site of infection and the age of the biofilm. At the beginning of infection when the biofilm is not fully established and bacteria remain genetically unchanged, antibiotic therapy even at a sub-optimal dose can prohibit the expansion of the bacterial population, allowing time for the immune system to resolve the infection. However, later in the infection when the biofilm is more established, immune mechanisms will eventually fail to control the infection and lead to chronic inflammation. It has been observed that lengthy antibiotic therapy may select for strains that produce high levels of persister cells over time [117], probably in parallel to the maturation of biofilm. Recent transcriptomic analyses show that bacteria respond to sub-lethal doses of antibiotics with a more generalized stress response mechanism rather than a specific response to a single antibiotic, and expression of virulence factors may be repressed accordingly [60,118]. Although these results were obtained from *in vitro* biofilms and cannot be directly applied to *in vivo* situations, such studies provide important insight into the mechanisms of the bacterial response towards sub-lethal concentrations of antibiotics and lay the foundation for future *in vivo* studies.

In trying to combat persisters, several strategies have been proposed. As early as 1944, Bigger proposed a pulse-dosing regimen to eradicate persisters [119]. His strategy was to kill bacteria with a high dose of an antibiotic, then allow the antibiotic concentration to drop, which enabled persister cells to come out of dormancy and start to divide. Eradication of bacteria may be achieved by a second dose of antibiotic administered shortly after persisters begin to divide. This approach was successful *in vitro*, and a *P. aeruginosa* biofilm was successfully eradicated with two consecutive administrations of a fluoroquinolone [87]. Unfortunately, this idea was not further assessed by *in vivo* models or clinical studies, probably partially due to the concern of selection pressure on resistant strains by the high dose of antibiotics. However, it is now clear that not only high doses but also sub-MIC doses of antibiotics can select for resistant strains [7,120]. Further studies are thus warranted to investigate the optimal dose and interval for the pulse-dosing regimen using *in vivo* models in addition to careful comparison of the collateral damage that different regimens may bring about. As also pointed out by Lewis [87], many chronic infections are eventually cured after long-term antibiotic therapy, analysis of these patients’ clinical outcomes together with the bacterial response (at genomic, transcriptomic and proteomic levels) over the course of antibiotic therapy may give insight into the mechanisms by which chronic infections are cured. Indeed, data in cystic fibrosis patients have shown that although *P. aeruginosa* tend to acquire and accumulate multiple antibiotic resistance over repeated antibiotic therapies, the increased antibiotic resistance is not necessarily to be associated with poorer outcomes in cystic fibrosis patients [121]. Similarly, infections with antibiotic-susceptible strains of *P. aeruginosa* are not always associated with improved outcomes in cystic fibrosis patients [122].

In addition to strategies maximizing the effect of currently available antibiotics, new techniques have been developed in the past few years utilizing strategies to prevent the formation of persisters, to kill dormant persisters, or to first awaken the persisters then kill them (see review article [113]). Persister cells have evolved over billions of years, fine-tuning genetic regulation through exposure to a huge array of harmful compounds from their environments and hosts. It is most likely that successful treatment will only be achieved by combined strategies [123].

## 6. Summary and Future Perspectives

Persisters in the biofilm are tolerant to several bactericidal antibiotics. Recent studies have shown that there are several mechanisms for tolerance development including through a decreased production of reactive oxygen species in the persister cells [124,125], by inhibition of macromolecule synthesis by toxin–antitoxin modules [126], or by reduction of cellular metabolism through the PhoU protein [127]. Although antibiotic tolerance in persisters is suggested to be phenotypic, antibiotic tolerant persisters may acquire mutations and develop genetically based resistance. Similarly, a genetically antibiotic resistant mutant could also develop persisters with tolerance. Therefore, genetic resistance and tolerance may interconvert and overlap [128,129]. For further understanding of persister mechanisms, development and selection of adequate persister models will be important. It is also difficult to obtain an accurate persister model since *in vitro* persisters are not the same as *in vivo* persisters due to differences in the environments that the bacteria reside in and the presence or absence of antibiotic exposure. Although there is progress in our knowledge of persister development in recent years, the detailed mechanism of persisters remains to be studied. With the application of the “omics” approaches (transcriptome, proteome, metabolome, and epigenome) and next-generation sequencing techniques to analyze the persisters, this research field will continue to develop rapidly in the near future.

The biofilm-associated bacteria have evolved increased capacity to tolerate antibiotics comparing to their planktonic counterparts, hampering the successful treatment of biofilm-associated infections. Strategies to combat biofilm infection include early diagnosis of biofilm-associated infections, novel strategies to prevent formation of biofilm, and strategies to remove biofilm when they are formed. These aspects have been covered by several recent review articles [1,112,130,131]. Deeper understanding of antimicrobial resistance resulted from the simultaneous operation of multiple biofilm-specific mechanisms will be helpful for eradication of biofilm-associated persistent infections. Particularly, studies on biofilms isolated from *in vivo* and clinical sources, by using genome sequencing, comparative genomics, proteomics and RNA-sequencing, have opened the door for developing novel anti-biofilm agents. Here we add a few new anti-biofilm strategies that were reported during the past one year, emphasizing mechanisms involved in bioelectric effect [132], membrane vesicles [133] and eDNA [134].

A combinational treatment utilizing low doses of antibiotics and electrical signals, also termed as “bioelectric effect”, has been shown to be effective in biofilm treatment [135,136]. In addition to traditional cell-to-cell communication systems such as quorum sensing, bacteria can use electron flux to communicate [137]. Recently, an elegant study by Prindle *et al*. [132] demonstrated that bacteria use potassium ion-channel-mediated long-range electrical signaling to coordinate metabolism within the biofilms. The waves of charged ions in the biofilms coordinated the metabolic activity of bacteria in the inner and outer regions of the biofilms. When the gene encoding a potassium ion-channel, *yugO*, in *Bacillus subtilis* was deleted from the bacteria, the biofilm was no longer able to conduct these electrical signals leading to impairment of biofilm formation. Therefore, challenges to develop anti-biofilm drugs that target potassium ion-channels are offering considerable opportunities for future success.

Recent findings indicate that secreted protease/peptidases may play a role in *V. cholerae* biofilm formation [138,139]. These proteases are shown to be secreted from the bacterial cells in association with bacterial membrane vesicles (BMVs) [81,140]. BMVs are involved in both the delivery of toxins and for cellular communications between bacteria within a biofilm. Interestingly, a recent proteomic study demonstrated that BMVs from the bacterial predator *Myxococcus xanthus* carried not only a high amount of proteases and peptidases (11 identified in the BMVs), but also a putative chitinase, phosphoesterase, hydrolase and nuclease [141]. Moreover, the relative abundance of the proteins show an enrichment of the BMVs with lipoproteins, hydrolases and the chaperonin GroEL, compared to that of whole cells and the outer membrane fraction. Analysis of the secondary metabolites indicate the presence of antimicrobials in the BMVs, e.g. the cittilin A, the myxovirescin A and the myxalamid A, B and C [142]. The myxovirescin inhibits the type II secretion system and the myxalamids inhibit the cytochrome I NADH:ubiquinone oxidoreductase. The target of the cittilin A remains unknown. Considering the key roles of BMV-associated bacterial proteases for recruitment/reinforcement of biofilms, BMVs could present interesting targets in new efforts for anti-biofilm treatment [133].

eDNA has been found to promote biofilm formation of many different bacterial species and has been suggested to be a promising new target for anti-biofilm treatments. Removal of eDNA could lead to destabilization of biofilms and increase the antibiotic and biocides susceptibility. By targeting eDNA in biofilms, commercially available bovine or human DNase I has been used to treat several bacterial biofilm infections [134]. It is often observed that eDNA in the biofilm matrix was not efficiently accessible to the enzyme since it was embedded in other matrix components such as exopolysaccharides and matrix proteins. It is therefore necessary to take into consideration how eDNA interacts with other matrix components and whether DNA-binding proteins may be present in the biofilm matrix. Such bacterial DNA-binding proteins might also be potential targets for anti-biofilm treatment.

There is a need for new methods to monitor the effect/response of biofilm infection towards antibiotic therapy. Current routine MIC tests, from disc diffusion to microdilution-based automated methods, are performed with planktonic bacteria under standardized *in vitro* conditions. First, results obtained from such *in vitro* conditions may not reflect the biofilm-forming ability and the effective antibiotic concentrations *in vivo* [143,144]. Different *in vitro* conditions may give different results even performed on the same isolate [145]. Second, compared to planktonic bacteria, biofilm bacteria often exhibit increased tolerance to antibiotics as discussed above. In order to solve the problem, some susceptibility tests have been designed to examine the MIC of bacteria associated with biofilms, for example, the Calgary device that is used to grow biofilms on pegs protruding from the lid of a microtiter plate and subsequently used to expose the biofilms to various concentrations of antibiotics [146]. However, such tests have not yet shown a reliable prediction of therapeutic success, reflecting the complexity of *in vivo* biofilms in comparison with *in vitro* biofilms [147]. These issues make it difficult to compare the different published studies using various methods and are probably the reason why some studies found an association between biofilm-forming bacteria and more severe clinical outcomes [110,148] while others did not [149]. Successful treatment of biofilm infections requires a multidisciplinary collaboration between clinical microbiology, surgery, internal medicine, pharmacology and basic science.
